# Reproductive health challenges of an African school girl: a case report on non-bulging imperforate hymen with haematocolpometra during Covid-19 pandemic

**DOI:** 10.4314/ahs.v23i3.16

**Published:** 2023-09

**Authors:** Raymond Bvumbi, Nnabuike Chibuoke Ngene

**Affiliations:** 1 Department of Obstetrics and Gynaecology, School of Clinical Medicine, Faculty of Health Sciences, University of the Witwatersrand, Johannesburg, South Africa; 2 Department of Obstetrics and Gynaecology, Leratong Hospital, Krugersdorp, Gauteng, South Africa

**Keywords:** African schoolgirl, cryptomenorrhea, haematocolpometra, hydrometrocolpos, impact of COVID-19, non-bulging imperforate hymen

## Abstract

**Background:**

Several schoolgirls attain reproductive age with undiagnosed gynaecological problems which pose challenges in their livelihood. These conditions include precocious puberty, congenital reproductive tract abnormalities, and delayed sexual development. Many children with these conditions face additional challenges including physical pain, psychological trauma and delayed diagnosis.

**Methods:**

A 14-year-old girl presented with acute on chronic pelvic pain and haematocolpometra due to imperforate hymen during COVID-19 pandemic. She has not undergone cultural virginity test in her community. The hymenal membrane was unusually non-bulging despite the haematocolpometra. A partial hymenotomy with a narrow margin of excision was performed.

**Results:**

The hymenal orifice later obliterated and resulted in a repeat partial hymenectomy where a wide surgical margin of the hymen was excised.

**Conclusions:**

A wide rather than narrow partial hymenectomy prevents obliteration of the hymenal orifice after surgery for imperforate hymen. There is a need for timely interventions such as counselling and community awareness that prevent undue consequences of an imperforate hymen and its treatment including pain and possible inability to pass cultural virginity test in some African communities.

## Introduction

Coronavirus Disease 2019 (COVID-19) pandemic which is caused by severe acute respiratory syndrome corona-virus 2 (SARS-CoV-2) has resulted in the death of over 2.68 million people worldwide, as at 3:44 pm Central European Time, 19 March 2021.^1^ The disease is highly contagious and resulted in nation-wide lockdown in many countries with the restriction of access to public places to contain the spread of the infection.^2^ Such restrictions were also applied to healthcare facilities in South Africa to reserve and prepare medical resources for patients who become sick with the COVID-19 infection. The restrictions delayed the medical treatment of ‘non-urgent’ clinical conditions.^2^ The index case is on non-bulging imperforate hymen in the presence of hydrometrocolpos which has not been previously reported. In the case report, the authors illustrate the undue consequences of delayed presentation of non-bulging imperforate hymen during COVID-19 pandemic. The effectiveness of wide partial hymenectomy for treating imperforate hymen and the impact of such procedure on cultural virginity testing in Africa are also explored.

## Case presentation

A 14-year-old female pupil who is HIV positive from vertical transmission and on effective antiretroviral drug therapy presented to a primary healthcare clinic during COVID-19 pandemic with a 4-month history of cyclical lower abdominal pain which became acute two days before arrival. She was referred to a tertiary hospital as a case of ovarian torsion.

**Figure 1:**
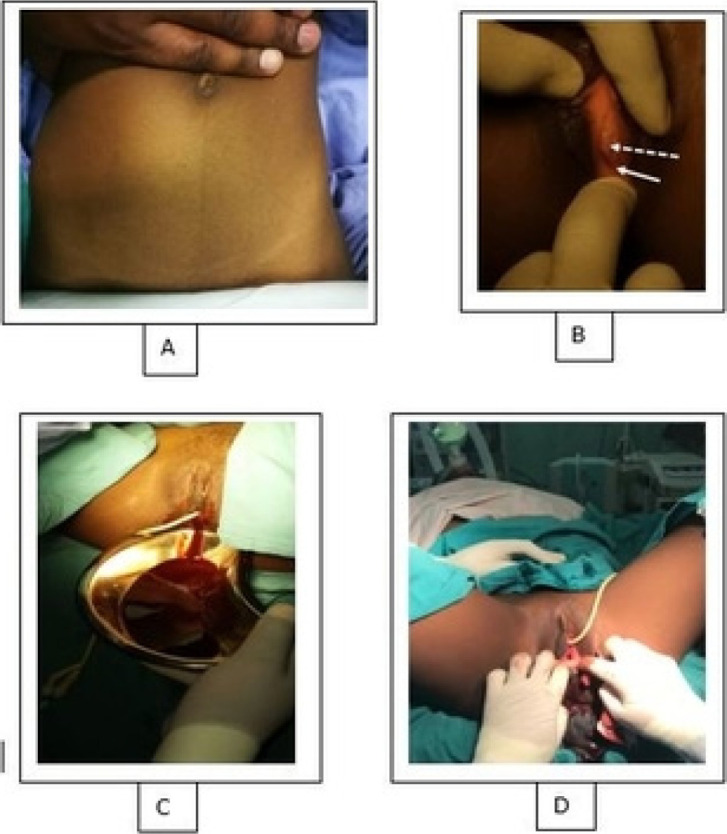
Twenty weeks uterine size abdominopelvic mass (A), urethra (dashed arrow) and non-bulging imperforate hymenal membrane (solid arrow) despite the presence of haematocolpos with the image also showing a short distance between the urethra and the hymen (B), drainage of haematocolpometra following hymenectomy (C), and hymenal orifice after second hymenectomy, (D)

On arrival to the tertiary hospital, she had severe abdominal pain, backache, constipation and was yet to experience menarche or coitarche. The patient had little reproductive health knowledge, initially thought that the abdominal pains were normal and did not seek medical help due to COVID-19 restrictions. The vital signs were normal. She had an abdominopelvic mass of 20 weeks uterine size which was tender with associated abdominal guarding. Perineal examination revealed an imperforate non-bulging hymen (Figure 1) and no other obvious congenital abnormality. Ultrasonography showed distended uterus and vagina filled with fluid suspected to be blood. The patient and her mother were counselled, and they consented to hymenectomy. Analgesic was administered. Pre-operative evaluation including full blood count and renal functions were normal. Under general anaesthesia in a lithotomy position, narrow partial hymenectomy was performed because of the short distance between the external urethral meatus and the small-diameter hymen and 300 ml of altered blood was drained intraoperatively. Post-operatively, the patient was counselled and discharged home. On post-operative day 29, she presented with mild lower abdominal pain with no visible orifice on the hymenal membrane. She underwent wide partial hymenectomy (using a cruciate incision) to drain haematocolpometra (Figure 1) and had a normal postoperative follow-up.

## Discussion

Imperforate hymen is a rare congenital abnormality of the female genital tract where the hymen completely obliterates the vaginal orifice. It is caused by the non-canalization of the distal end of the vaginal plate during embryogenesis. The prevalence is 0.05 - 0.1% amongst girls.^3^-^5^ It is usually diagnosed at puberty when girls are expected to start menstruating. Because there is outflow obstruction, the menstrual blood accumulates in the vagina (haematocolpos) and uterus (haematometra)^3^, ^4^ causing the hymenal membrane to be bluish and bulging as shown in previously published images.^6^

These patients usually present with cyclical abdominal pain and as the fluid accumulates, symptoms worsen and the mass-effect may cause constipation and urinary tract complications.^3^, ^5^ In the index case, the patient had cyclical lower abdominal pain which was associated with constipation, backache, abdominopelvic mass and non-bulging hymenal membrane. The lack of obvious bulging of the hymenal membrane despite the presence of haemato-colpos (Figure 1) makes this case unique and most likely was due to the increased thickness of the hymen. The patient reported the symptoms late to her parents because of little reproductive health knowledge which made her thought that the symptoms were normal. During the peak of COVID-19 pandemic, most countries imposed nation-wide lockdown and many patients could not seek medical help for “non-urgent” conditions,^2^ and this could have contributed to the delay in the patient seeking healthcare. Other investigators have opined that the impact of COVID-19 pandemic on vulnerable individuals is far-reaching.^7^ Understandably, adolescents with undiagnosed imperforate hymen will usually present to healthcare facilities for the first time after menarche following the onset of abdominal pain from cryptomenorrhea, although the initial complaint by some patients may be the inability to have penetrative sexual intercourse.

In many Africa settings, there is limited reproductive healthcare knowledge amongst pupils and insufficient school health surveillance which results in delayed diagnosis of imperforate hymen^4^. The delayed diagnosis results in undue suffering before diagnosis. Due to the cyclical pains, they miss school, and this may have an impact on their academic performance. When the diagnosis is finally made, the trauma of the revelation adds to the emotional suffering which also affects the family. The condition is also associated with the psychological trauma of not having periods at an expected age^4^.

Typically, the management of patients with imperforate hymen involves hymenotomy or hymenectomy to drain the uterine and vaginal contents to relief the pain and pressure symptoms.^5^,^8^ Following the surgical drainage (particularly after a simple hymenotomy), obliteration of the hymen may occur and the patient will require further intervention.^8^ This complication occurred in the index case most likely due to a small amount of hymenal tissue excised during the initial partial hymenectomy. Other complications arising from untreated cases include chronic pelvic pain, subfertility, infections, endometriosis, hydronephrosis and renal failure.^5^, ^8^

In some communities, young girls go for virginity testing^9^-^11^ before marriage and if they fail this test, it might signify promiscuity resulting in being treated as a social outcast despite the discourse that virginity testing violates human right and may not be reliable in detecting previous penetrative intercourse. Therefore, some patients and their parents would like to preserve virginity by avoiding hymenectomy^8^ as this will have a cultural impact on their community. These girls and their caregivers also worry about sexuality and future fertility hence proper counselling is required pre- and post-operatively. Community awareness is also necessary to prevent social maltreatment of the affected girls and their families (Table 1).

**Table 1:** Highlights

S/No	Points
1	A major collateral issue of the COVID-19 pandemic is delayed diagnoses of non-COVID-19 conditions.

2	Non-bulging imperforate hymen despite the presence of hydrometrocolpos has not been previously reported and is a very rare gynaecological presentation associated with delayed diagnosis.

3	Partial hymenectomy where a wide (rather than narrow) margin of the hymen is excised prevents hymenal obliteration after surgery for imperforate hymen.

4	Hymenectomy may result in a girl failing a cultural virginity test, and there is a need to counsel the patient and her family as well as to create community awareness to discuss the appropriateness of this cultural practice.

## Conclusion

To improve early diagnosis of imperforate hymen and reduce the associated co-morbidities, an examination of the neonate at birth is mandatory. Healthcare professionals should also examine adolescent girls that present with abdominal pain and amenorrhoea to exclude reproductive tract abnormalities such as imperforate hymen. Health education on reproductive tract should be emphasised at schools and any girlchild with suspected abnormalities must be referred early for appropriate assessment and management. A wide rather than narrow partial hymenectomy prevents obliteration of the hymen orifice after surgery. Psychological support also forms part of the management of such cases as these patients are physically and emotionally affected by the condition. A policy that enables school-going children to report ailments and safely access healthcare facilities for painful symptoms during a pandemic such as COVID-19 infection is recommended.

## References

[1] World Health Organisation WHO Coronavirus Disease (COVID-19) Dashboard.

[2] Hendrikse C, Parak M, van Hoving DJ (2020). A descriptive analysis of the effect of the national COVID-19 lock-down on the workload and case mix of patients presenting to a district-level emergency centre in Cape Town, South Africa. S Afr Med J.

[3] Lee KH, Hong JS, Jung HJ, Jeong HK, Moon SJ, Park WH (2019). Imperforate Hymen: A Comprehensive Systematic Review. J Clin Med.

[4] Posner JC, Spandorfer PR (2005). Early detection of imperforate hymen prevents morbidity from delays in diagnosis. Pediatncs.

[5] Shaw LM, Jones WA, Brereton RJ (1983). Imperforate hymen and vaginal atresia and their associated anomalies. J R Soc Med.

[6] Stone SM, Alexander JL (2004). N Engl J Med Imperforate Hymen with Hematocolpometra.

[7] Hutchinson-Colas J, Sachdev D (2021). COVID-19 and pregnancy care for incarcerated women. Case Rep Womens Health.

[8] Cetin C, Soysal C, Khatib G, Urunsak IF, Cetin T (2016). Annular hymenotomy for imperforate hymen. J Obstet Gynaecol Res.

[9] Crosby SS, Oleng N, Volpellier MM, Mishori R (2020). Virginity testing: recommendations for primary care physicians in Europe and North America. BMJ Glob Health.

[10] Olson RM, García-Moreno C (2017). Virginity testing: a systematic review. ReprodHealth.

[11] Rakubu M (2019). The practice of virginity testing in South Africa: a constitutional and comparative analysis.

